# In Situ Raman Spectroscopy Reveals Structural Evolution and Key Intermediates on Cu-Based Catalysts for Electrochemical CO_2_ Reduction

**DOI:** 10.3390/nano15191517

**Published:** 2025-10-03

**Authors:** Jinchao Zhang, Honglin Gao, Zhen Wang, Haiyang Gao, Li Che, Kunqi Xiao, Aiyi Dong

**Affiliations:** 1 Marine Engineering College, Dalian Maritime University, Dalian 116026, China; zhangjingchao@dlmu.edu.cn (J.Z.);; 2 Hangzhou Dahua Apparatus Manufacture Co., Ltd., Hangzhou 310024, China; 3 School of Science, Dalian Maritime University, Dalian 116026, China

**Keywords:** in situ Raman spectroscopy, CO_2_RR, Cu-based catalysts, structural evolution, reaction intermediates

## Abstract

Electrochemical CO_2_ reduction reaction (CO_2_RR) is a key technology for achieving carbon neutrality and efficient utilization of renewable energy, capable of converting CO_2_ into high-value-added carbon-based fuels and chemicals. Copper (Cu)-based catalysts have attracted significant attention due to their unique performance in generating multi-carbon (C_2+_) products such as ethylene and ethanol; however, there are still many controversies regarding their complex reaction mechanisms, active sites, and the dynamic evolution of intermediates. In situ Raman spectroscopy, with its high surface sensitivity, applicability in aqueous environments, and precise detection of molecular vibration modes, has become a powerful tool for studying the structural evolution of Cu catalysts and key reaction intermediates during CO_2_RR. This article reviews the principles of electrochemical in situ Raman spectroscopy and its latest developments in the study of CO_2_RR on Cu-based catalysts, focusing on its applications in monitoring the dynamic structural changes of the catalyst surface (such as Cu^+^, Cu^0^, and Cu^2+^ oxide species) and identifying key reaction intermediates (such as *CO, *OCCO(*O=C-C=O), *COOH, etc.). Numerous studies have shown that Cu-based oxide precursors undergo rapid reduction and surface reconstruction under CO_2_RR conditions, resulting in metallic Cu nanoclusters with unique crystal facets and particle size distributions. These oxide-derived active sites are considered crucial for achieving high selectivity toward C_2+_ products. Time-resolved Raman spectroscopy and surface-enhanced Raman scattering (SERS) techniques have further revealed the dynamic characteristics of local pH changes at the electrode/electrolyte interface and the adsorption behavior of intermediates, providing molecular-level insights into the mechanisms of selectivity control in CO_2_RR. However, technical challenges such as weak signal intensity, laser-induced damage, and background fluorescence interference, and opportunities such as coupling high-precision confocal Raman technology with in situ X-ray absorption spectroscopy or synchrotron radiation Fourier transform infrared spectroscopy in researching the mechanisms of CO_2_RR are also put forward.

## 1. Introduction

Over the past century, the large-scale use of fossil fuels has greatly promoted industrialization and the rapid development of modern society, and promoted the progress of human civilization. However, this dependence is contributing to global climate change and environmental degradation caused by greenhouse gas (especially CO_2_) emissions; it also leads to the continued depletion of fossil resource reserves [1,2]. According to statistics, human activities emitted almost 37.8 ± 3.0 billion tons of CO_2_ into the atmosphere in 2023, and this number continues to rise [3]. To mitigate global warming and develop sustainable energy systems, the conversion of CO_2_ into high-value-added chemicals or fuels has become a focus of global scientific research and industry [4,5,6]. Among numerous CO_2_ conversion technologies, the electrocatalytic CO_2_ reduction reaction (CO_2_RR) stands out due to its advantages such as operation under mild conditions, direct driving by renewable energy, and tunable product types; it primarily reduces to single-carbon products (C_1_) or multi-carbon products (C_2+_) [7]. Compared with C_1_, C_2+_ has attracted much attention due to its higher energy density and economic value; however, in its formation process, it usually involves complex multi-step proton-coupled and multi-electron transfer reactions, which will result in complex reaction mechanisms and challenging selective regulation [8]. This technology not only provides a feasible approach for the resource utilization of CO_2_, but also can balance greenhouse gas emission reduction and renewable energy storage; thus, it has important strategic significance in achieving carbon neutrality goals and building a sustainable energy system [9,10,11,12].

The CO_2_RR process involves multiple electron-proton coupling reactions; for example, its theoretical thermodynamic potential for the CO_2_ to CO reduction reaction is −0.106 V vs. RHE, but due to the complexity of reaction kinetics, in actual operation, it is often necessary to apply an additional overpotential to overcome electrode polarization loss [13]. Compared with C1, which usually only requires fewer electron transfer steps to form, the formation of C_2+_ involves multiple consecutive electron–proton transfers and C-C coupling processes, and the reaction energy barriers of these steps are significantly higher than those of the general C_1_ product pathway [14,15]. This leads to the kinetics of C_2+_ product formation being significantly slower than that of C_1_ products, thus becoming a key factor limiting the improvement of overall selectivity. Therefore, to improve the C_2+_ product selectivity and reaction rate of CO_2_RR, there is an urgent need to develop electrocatalysts with high activity and high selectivity under low overpotential. Au and Ag have moderate adsorption energy for the key intermediate *COOH, thus exhibit excellent catalytic activity and high Faradaic efficiency in the CO_2_ to CO conversion, and are widely regarded as benchmark materials for CO production via CO_2_RR. Among them, Au catalysts usually exhibit higher intrinsic activity, while Ag has advantages in inhibiting the hydrogen evolution reaction, and both excel in the selective production of CO products [16]. However, such metals are scarce in reserves, expensive in price, and it is difficult for them to effectively generate multi-carbon products, which severely limits their prospects in large-scale applications.

Cu-based catalysts, due to their unique electronic structure and moderate CO adsorption energy, have become ideal candidate materials for C_2+_ production via CO_2_RR. Studies have shown that under the synergistic effect of Cu^0^–Cu^+^ and orbital hybridization, the adsorption of intermediates (such as *CO, *OCCO) can be enhanced, and the energy barrier of C–C coupling reactions can be reduced, thereby promoting the formation of C_2+_ products [4,17,18,19,20,21,22,23]. Compared with noble metals, which mainly produce C_1_, Cu has the advantages of abundant reserves, low cost, and being more conducive to the formation of multi-carbon products. Moreover, its catalytic performance can be further optimized through methods such as oxide derivation, alloying, and surface structure regulation. The designability of Cu-based crystal structures and surface morphologies enables it to expose more active sites and optimize intermediate adsorption through methods such as constructing high-index crystal planes, introducing interface defects, and regulating nanostructures, thereby significantly improving the selectivity and Faraday efficiency(FE) of C_2+_ products [4,24,25]. However, in recent years, studies have pointed out that under CO_2_RR conditions, Cu^δ+^ species and surface oxygen/hydroxyl groups (such as CuO_x_, CuO_x_(OH)_γ_) may play a role in C_2+_ formation, but this conclusion still requires joint verification by multiple technical methods [26].

In recent years, to deeply reveal the structural evolution and active sites of Cu-based catalysts in CO_2_RR, the importance of in situ characterization techniques has become increasingly prominent. Compared with traditional ex situ analysis, in situ methods can capture the real-time dynamics of catalysts in the real reaction environment, including key processes such as structural reconstruction of catalysts and formation, transformation, and disappearance of intermediates. Among them, in situ Raman spectroscopy, due to its high sensitivity to surface molecular vibrations and “fingerprint recognition” capability, performs prominently in detecting catalyst surface structures and identifying reaction intermediates. Meanwhile, in aqueous electrolyte environments, in situ Raman can effectively reduce the strong absorption interference from water molecules, and compared with in situ Fourier transform infrared spectroscopy (FTIR), it is more suitable for the research on CO_2_RR. With in situ Raman, researchers have successfully detected key C_2+_ intermediates such as *OCCO and *CH_2_CHO, and combined with the potential dependence of different crystal planes (such as Cu(110) or Cu(111)), identified the C_1_/C_2_ product formation pathways [27]. Another key study employed Sub-second Time-Resolved Surface-Enhanced Raman Spectroscopy (TR-SERS) technology, with a time resolution of approximately ~0.7 s per frame, to reveal the fast kinetic behavior of adsorbed *CO intermediates. The results indicate that C–C coupling leading to ethylene formation and the formation of highly reactive *CO species adsorbed below 2060 cm^−1^ are strongly correlated, while the relatively stable *CO species at approximately 2090–2095 cm^−1^ observed under lower cathodic bias mainly correspond to the formation pathway of gaseous CO [28,29,30]. The introduction of signal amplification strategies such as Surface-Enhanced Raman Spectroscopy has significantly improved the detection sensitivity of low-coverage and transient intermediates, enabling in situ Raman to play a critical role in revealing the structural evolution of Cu catalysts and reaction intermediates.

Therefore, this review focuses on the structural evolution and analysis of active sites of Cu-based catalysts in CO_2_RR, systematically discusses the latest progress of in situ Raman spectroscopy in the field of CO_2_RR, with emphasis on summarizing the role of in situ Raman spectroscopy in revealing the conversion behavior of Cu^0^, Cu^+^ and Cu derivative species, as well as identifying key intermediates such as *CO, *OCCO, *COOH, *CH_2_CHO, etc and providing new ideas and methods for the design of efficient C_2+_ product electrocatalytic conversion systems.

## 2. Basic Principles and Construction of Electrochemical In Situ Raman Spectroscopy

Changes in gas and liquid interfaces, electrolytes, and overpotentials lead to variations in intermediates or products. This process involves multiple steps of proton coupling and electron transfer. Even small changes in the activation energy for each step can significantly affect the Faraday efficiency of the products. The application of in situ spectroscopic techniques to elucidate the fundamental mechanisms of CO_2_ reduction reactions facilitates the rational design and development of catalysts [31]. Raman spectroscopy is based on the inelastic scattering of light with matter (anti-Stokes and Stokes scattering), which triggers energy exchanges between photons and molecular or lattice phonon vibrations, resulting in a frequency shift (Δν) of the scattered light. Consequently, the energy changes of scattered photons (molecular vibration modes) provide valuable information about the chemical composition, molecular structure, and bonding characteristics of the sample [32].

**Figure 1:**
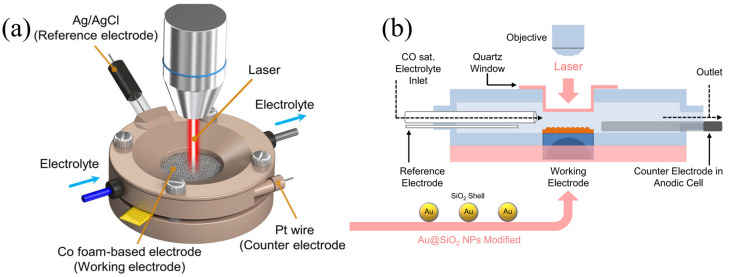
(**a**) In situ Raman spectroscopy apparatus. Reproduced with permission from Ref. [33]. Copyright 2022, Nature Publishing Group. (**b**) Schematic of the flow cell for in situ SERS test. Reproduced with permission from Ref. [34]. Copyright 2022, Nature Publishing Group.

In situ Raman spectroscopy has the characteristics of Raman spectroscopy and electrochemistry, and realizes the dynamic analysis of catalyst surface structure, electrode/electrolyte interface intermediate evolution and reaction path. Figure 1a shows a schematic diagram of the electrochemical/Raman spectroscopy system, demonstrating its important role in identifying key information [33]. Thus, it provides molecular-level mechanistic insights into complex electrochemical interfaces. Weak signal intensity, laser-induced sample damage, and background fluorescence interference remain core technical challenges for electrochemical in situ Raman spectroscopy. These can be mitigated by optimizing energy transfer efficiency along the photon transmission path, adjusting laser parameters (wavelength, power, and exposure time), designing optical windows and interfaces, selecting appropriate optical window materials, and minimizing the distance between the optical window and the electrode. In electrochemical environments, the Raman enhancement effect is crucial for a comprehensive understanding of materials and phenomena. Among various Raman enhancement techniques, SERS has become a focal point, with metals such as copper (Cu), silver (Ag), and gold (Au) used to prepare SERS substrates that can enhance Raman signals by 6–10 orders of magnitude [34,35,36]. For CO_2_RR, the nanoscale rough metal surfaces of copper catalysts exhibit significant surface-enhanced Raman spectroscopy effects, making them ideal SERS substrates. For atomically flat copper surfaces (e.g., foils or single crystals), shell-isolated nanoparticle-enhanced Raman spectroscopy (SHINERS) addresses the limitations of traditional SERS in terms of morphology and material versatility, with studies confirming its feasibility [37,38]. Chang et al. developed an in situ spectroscopic cell and procedure shown in Figure 1b, illustrating a flow cell setup for SERS testing, integrated with an SEIRAS cell to enable tandem in situ characterization. This SERS setup effectively analyzes surface species (e.g., CO adsorption configurations) and identifies reaction intermediates, offering a spectral window down to tens of wavenumbers, covering vibrational modes inaccessible to SEIRAS. With low water sensitivity and chemical enhancement effects, it distinguishes species in different adsorption environments, complementing Surface-Enhanced Infrared Absorption Spectroscopy (SEIRAS), particularly on weakly adsorbing metal surfaces such as Au and oxide-derived copper (OD-Cu), thereby detecting distinct adsorbed species. This approach provides critical information on reaction intermediates for electrocatalytic reaction mechanism studies [34,36].

## 3. Reaction Pathways in the CO_2_RR Process

**Figure 2:**
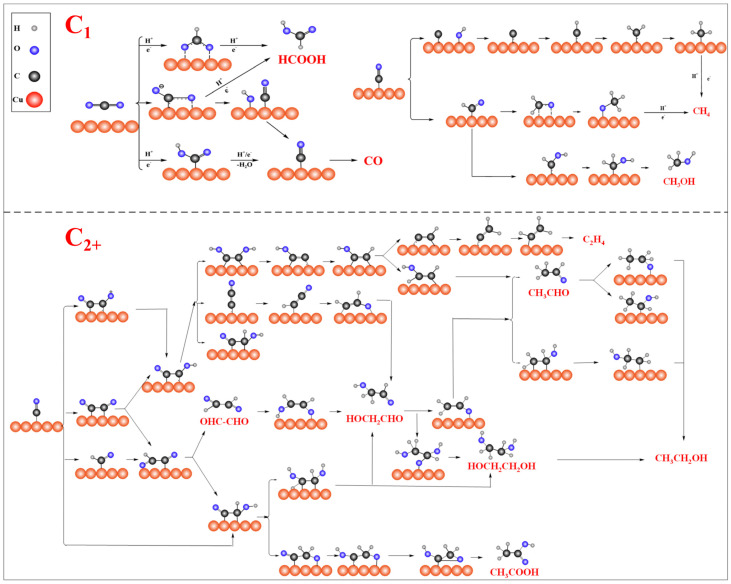
Proposed reaction pathways of CO_2_RR over Cu − based catalysts.

CO_2_RR can convert CO_2_ into value-added chemical fuels powered by clean energy, such as renewable solar or wind energy, and is currently a promising solution to alleviate the growing greenhouse gas emissions and energy crisis [39,40,41,42]. As shown in the reaction pathway diagram in Figure 2, due to the involvement of multiple proton-coupled electron transfer (PCET) processes in the reaction, a variety of products (C_1_/C_2+_) can be generated, including carbon monoxide (CO), Methane (CH_4_), methanol (CH_3_OH), formic acid (HCOOH), ethylene (C_2_H_4_), ethanol (CH_3_CH_2_OH), Acetic Acid (CH_3_COOH) and many other products.

**Figure 3:**
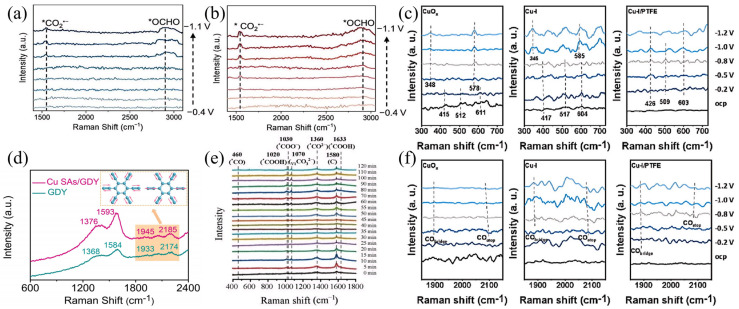
In situ Raman spectra recorded at different potentials ranging from −0.4 to −1.1 V vs. RHE for (**a**) pure Bi and (**b**) bimetallic Cu−Bi nanostructures. Reproduced with permission from Ref. [44]. Copyright 2025, Elsevier. (**c**) 300−730 cm^−1^ on CuO_x_, Cu−I, and Cu−I/PTFE in CO_2_−saturated 0.5 M KHCO_3_ electrolytes at various potentials. Reproduced with permission from Ref. [46]. Copyright 2023, Elsevier. (**d**) Raman spectra of pristine GDY and Cu SAS/GDY. Reproduced with permission from Ref. [45]. Copyright 2023, Wiley−VCH GmbH. (**e**) In situ Raman spectroscopy obtained from CuCoAlFeNi HEA catalyst-based H−Cell. The data was collected every 5 min. Reproduced with permission from Ref. [47]. Copyright 2025, The Royal Society of Chemistry. (**f**) 1850−2150 cm^−1^ on CuO_x_, Cu−I, and Cu−I/PTFE in CO_2_−saturated 0.5 M KHCO_3_ electrolytes at various potentials. Reproduced with permission from Ref. [46]. Copyright 2023, Elsevier.

The formation of C_1_ products in the C_1_ pathway is synergistically regulated by the catalytic sites on Cu^0^/Cu^+^ surfaces and adsorbed intermediates such as *COOH and *OCHO [40]. Through the in situ Raman spectroscopy characterization method, the adsorption behavior, energy barriers of these intermediates, and their quantitative effects on the FE and selectivity of C_1_ products have been revealed, providing a theoretical basis at the molecular scale for the optimization of the structure-activity relationship of catalysts. Yang et al. designed a novel MOF-derived Cu@Cu_2_O heterogeneous electrocatalyst with moderate intermediate adsorption, which is used for highly selective reduction of CO_2_ to methanol [43]. They used Raman spectroscopy to analyze the surface functional groups of the catalyst. Spectra were obtained from −0.6 to −0.9 V vs. RHE with 32 consecutive scans. The observed characteristic band at 2350 cm^−1^ was attributed to the adsorption of CO_2_. The catalyst exhibits the highest CO_2_ peak intensity at −0.7 V. The peak at 1690 cm^−1^ may be the characteristic peak of *HCO_3_. The two new characteristic peaks observed at 1390 and 2065 cm^−1^ are attributed to *COOH and *CO groups. Due to the continuous consumption of CO_2_ during the catalytic reaction, the intensity of the two peaks gradually increases from −0.6 V to −0.9 V. Notably, a new band observed at approximately 1750 cm^−1^ is due to the presence of *CHO. As the catalyst potential gradually increases, the concentration of 1690 cm^−1^ (*HCO_3_) decreases, while the concentration of the intermediate (*CHO) increases. *CHO is a key intermediate in the electrocatalytic reduction of CO_2_ to methanol; thus, it is inferred that CO_2_ molecules on the adsorbed surface equilibrate with KHCO_3_, then convert to *CHO, and thus reduce to methanol [43]. Li et al. designed an electron structure manipulation strategy for electron-rich Cu-Bi nanosheets, in which electrons transfer from Cu donors to Bi acceptors in bimetallic Cu-Bi, endowing CO_2_RR with high activity, selectivity, and stability in pH-universal acidic, neutral, and alkaline electrolytes. They performed in situ Raman spectroscopy detection, applied a potential of −0.4 to −1.1 V vs. RHE on the HOD-Cu catalyst, and observed two vibrational peaks at 1640 and 2080 cm^−1^, which are assigned to *COOH and *CO intermediates. This indicates that a large amount of protonation of carbon atoms in CO_2_ occurs on the HOD-Cu electrocatalyst, forming HCOOH, CO, and C_2+_ products. For pure Bi nanosheets, as shown in Figure 3a, two distinct Raman peaks appear at 1530 and 2895 cm^−1^, and these peaks are attributed to the vibrational fingerprints of the asymmetric C−O stretching mode of proton-captured carboxylate CO_2_•− radicals, as well as the C−H stretching vibrations of *OCHO intermediates. For the bimetallic Cu-Bi electrocatalyst as shown in Figure 3b, the applied potential at which the vibrational peaks of the intermediates CO_2_•− (1538 cm^−1^) and *OCHO (2905 cm^−1^) appear is −0.6 V vs. RHE, significantly lower than the −0.9 V vs. RHE observed in the pure Bi environment. This confirms that the electron-rich Bi in bimetallic Cu-Bi significantly promotes the activation of CO_2_ molecules, facilitates the emergence of CO_2_•− intermediates, and is beneficial to the formation of formic acid [44]. Shi et al. designed the in situ anchoring of Cu SAs (single atoms) on graphdiyne, achieving the construction of the chemical bond Cu-C (GDY). Through in situ Raman spectroscopy, as observed in Figure 3d, the four peaks of pristine GDY at 1376, 1593, 1945, and 2185 cm^−1^ correspond to the vibrations of the D band, G band, and conjugated diyne bonds, respectively. Compared with pristine GDY, the peaks of the conjugated units in Cu SAs/GDY show a positive shift, which confirms that Cu-C bonds are formed after single Cu atoms are anchored on GDY. Due to the formation of Cu-C bonds, during CO_2_ reduction, mainly *OCHO intermediates rather than *COOH are formed on Cu atoms, which is beneficial to the formation of CH_4_[45]. Li et al. found in their study that activated Cu nanowires(A-CuNWs) exhibit a high proportion of Cu^+^/Cu^0^ active sites, and their surface can effectively enrich *CO intermediates and promote *CO_bridge_ adsorption, thereby enhancing C-C coupling to form *OCCO intermediates and finally generating C_2_H_4_. When the ionic liquid [Bmim][PF6] is introduced on the surface, the binding energy of Cu^+^/Cu changes, which increases the local proton donor density, thereby promoting the protonation of *CO to form *CHO and other key intermediates and weakening the C-C coupling process. The higher the proton donor density, the more the formation of C_2+_ products is inhibited, thereby generating CH_4_. The subtle regulation of Cu^+^/Cu valence states plays a crucial role in determining the adsorption mode of *CO intermediates and the subsequent reaction pathways [40].

In CO_2_RR, the formation of C_2+_ products usually involves complex C–C coupling and multi-electron–proton transfer processes, and their reaction pathways and intermediate stability are regulated by the electronic structure of active sites on the catalyst surface. In recent years, with the help of real-time characterization techniques such as in situ Raman spectroscopy, researchers have been able to directly analyze the adsorption configurations and energetic characteristics of key intermediates (e.g., *CO, *OCCO) under reaction conditions, revealing their influence on FE and product distribution of C_2+_ products. Xing et al. conducted real-time detection of the CO_2_RR mechanism of the CuCoAlFeNi HEA catalyst through in situ Raman measurements. Through the measurements, in situ Raman spectra (Figure 3e) were observed, with peaks appearing at 460 cm^−1^, 1030 cm^−1^, and 1360 cm^−1^, which belong to the vibrations of *CO, *COO^−^, and *CO^2−^. The C-O stretching of *COOH corresponds to the peaks at 1020 and 1633 cm^−1^. The peak at 1070 cm^−1^ can be attributed to the C-O stretching of adsorbed carbonate species (V_1_CO_3_^2−^). In the CO_2_RR process, CO_2_ undergoes electron transfer to form *COO^−^, and then generates *COOH through protonation. *COOH is easily reduced to *CO, and thus *CO can be further reduced to CH_3_CH_2_OH products through C-C coupling [47]. Zeng et al. constructed a hydrophobic microenvironment for CO_2_RR by coating the hydrophobic polymer polytetrafluoroethylene (PTFE) on the surface of Cu-I. And they used in situ Raman spectroscopy to investigate the possible CO_2_RR mechanism in CO_2_-saturated 0.5 M KHCO_3_ electrolyte. As shown in Figure 3c, Raman peaks around ~415, ~512, and ~611 cm^−1^ were observed, which belong to surface Cu^+^ species. The Raman peaks of CuO_x_ and Cu-I electrocatalysts disappear at −0.8 V vs. RHE, indicating that at high reduction potential, Cu+ is reduced to Cu0 due to poor stability. When the reduction potential ranges from −0.5 to −1.2 V vs. RHE, additional peaks around ~348 and ~578 cm^−1^ attributed to Cu-CO appear. In the Cu-I/PTFE electrocatalyst, these characteristic peaks (~426, ~509, and ~603 cm^−1^) remain unchanged during the test, indicating that the Cu species are reduced more slowly compared with the other two electrocatalysts. Furthermore, as observed in Figure 3f, the wavebands at 1850–1880 and 2050–2090 cm^−1^ are attributed to bridging CO (*CO bridge) and on-top CO (*CO atop). Compared with CuO_x_, the integrated area of the *CO band in Cu-I and Cu-I/PTFE increases, confirming that *CO has stronger binding to their surfaces. Since the hydrophobic surface plays a key role in stabilizing Cu^+^ species, the stable Cu^+^ species are considered to be active sites that promote CO_2_ activation and *CO dimerization, and the *CO intermediate is regarded as a key intermediate for the formation of C_2+_ products. Therefore, these data strongly demonstrate that hydrophobic Cu-I/PTFE can stabilize Cu^+^ species, thereby improving the activity and selectivity of CO_2_RR and increasing the production rate of C_2_H_4_ [46].

## 4. Detection of Reaction Intermediates Formed at Electrode/Electrolyte Interface

In CO_2_RR, the electrode/electrolyte interface (EEI) is the core region for reactant conversion and product formation, and its local environment exerts a determinative influence on reaction pathways and product distribution [48,49,50,51]. Interface processes usually involve complex reactions such as adsorption and activation of CO_2_ molecules, multiple electron–proton coupled transfer steps, and C–C coupling, during which various transient intermediates (e.g., *CO_2_, *COOH, *CO, *OCCO) are generated, and they are also hindered by the competition from the hydrogen evolution reaction (HER) [49,50]. Due to the low coverage and short lifetime of these intermediates at the interface, traditional ex situ characterization methods are difficult to capture their dynamic changes under real reaction conditions. SERS is highly sensitive to changes in molecular polarizability and can highly sensitively detect the vibrational modes of adsorbed species at the interface under electrochemical reaction conditions, thereby resolving the local chemical environment of EEI and the adsorption configurations of key reaction intermediates [48,51]. Additionally, verification through density functional theory (DFT) calculations not only reveals the effects of factors such as electrolyte composition and anion enrichment on the stability of intermediates and reaction pathways but also achieves a molecular-level understanding of the selectivity regulation of CO_2_RR.

**Figure 4:**
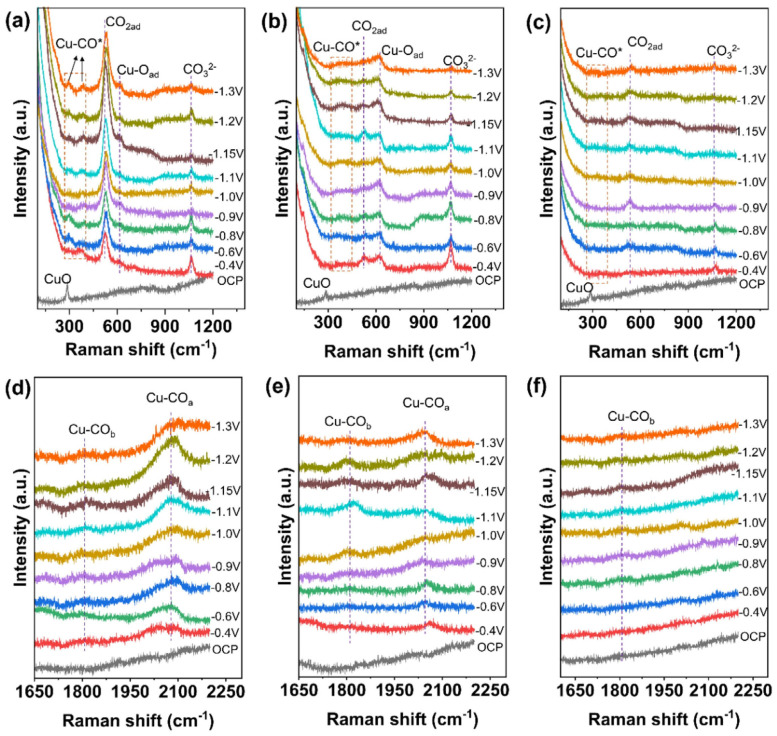
In situ Raman spectra of the catalysts under CO_2_RR conditions. (**a**,**d**) CuZn/CuZnAl_2_O_4_, (**b**,**e**) Cu/CuAl_2_O_4_, and (**c**,**f**) CuZn. The spectra were recorded at −1.15 V vs. RHE. All experiments were conducted in a 2 M KOH electrolyte under flow−cell conditions. Raman measurements were performed using a conventional visible laser source. Reproduced with permission from Ref. [59]. Copyright 2023, Elsevier.

*CO has been identified as an important and common intermediate in the evolution of high-value C_2+_ products (such as ethylene and ethanol), particularly on Cu-based catalysts [52]. However, due to the fact that the binding of CO to the catalyst surface usually does not involve significant charge transfer, it is difficult to detect signals of CO adsorption [53,54,55,56,57]. Three characteristic vibrational modes on the surface of Cu catalysts corresponding to Raman shift regions have been observed: Cu-CO rotation mode (280–290 cm^−1^), Cu-C stretching vibration mode (360–370 cm^−1^), and C≡O stretching vibration mode (1800–2100 cm^−1^). Lei et al. designed grain boundary-rich Cu nanoribbons on balanced gas-liquid diffusion electrodes for efficient CO_2_RR conversion to C_2_H_4_. They proposed that the thickness of the catalyst layer is a factor affecting CO_2_ bonding; by adjusting the thickness of the catalyst layer, CO_2_ and electrolyte mass transfer can occur simultaneously at the catalyst surface to participate in CO_2_RR. Raman shifts at 302 and 368 cm^−1^, belonging to the rotation and stretch modes of *CO on Cu (Cu-CO), were observed, confirming the adsorption of *CO intermediates and the formation of CO. C-C coupling forms *OCCO and *OCCOH intermediates, which improve the yield of C_2+_ products such as C_2_H_4_ [58]. Zhang et al. developed a Cu-Zn alloy/Cu-Zn aluminate oxide composite electrocatalytic system to enhance the conversion of CO_2_ to C_2+_ products. They investigated the oxidation state of Cu via in situ Raman spectroscopy. As shown in Figure 4, CuO species exhibit a sharp peak at 285 cm^−1^ under open circuit voltage, and this peak disappears rapidly when the cathodic voltage is −0.4 V, indicating that surface copper oxide is almost completely reduced to Cu^0^ species during the CO_2_RR process. The Raman peak at 529 cm^−1^ shown in Figure 4a is associated with the chemical adsorption of *CO_2_ on the copper surface, becoming more intense with the application of greater negative voltages. In contrast, as shown in Figure 4b,c, the *CO_2ad_ peak at 529 cm^−1^ on Cu/CuAl_2_O_4_ and CuZn surfaces decreases rapidly, indicating a low surface coverage of *CO_2ad_. They compared the Raman peaks at 529 cm^−1^ of CuZnAl_2_O_4_ samples and CuAl_2_O_4_ samples, confirming that the alloy/oxide interface significantly enhances the activation capacity of CO_2_. As shown in Figure 4a–c, the peaks at 297 and 375 cm^−1^ are attributed to the rotation and stretching modes of *CO on Cu (Cu-CO), indicating the formation of CO and the adsorption of *CO. Meanwhile, peaks in the range of 1800–1860 and 2000–2080 cm^−1^ observed in Figure 4d belong to *CO_bridge_ and *CO_atop_. However, Figure 4b,e show that the Cu-CO Raman signal becomes very weak on Cu/CuAl_2_O_4_, indicating a low coverage of CO, while on the pure CuZn alloy catalyst, this signal is hardly observed (Figure 4c,f). It can thus be inferred that the enhanced CO adsorption on CuZn/CuZnAl_2_O_4_ should be attributed to the CuZnAl_2_O_4_ oxide or positively charged Cu or Zn at the interface. These data demonstrate that the CuZnAl_2_O_4_ oxide at the interface enhances the adsorption of CO_2_ and CO, and the two adsorption modes of *CO_bridge_ and *CO_atop_ will promote C-C coupling with lower reaction energy barriers [59]. Jiao et al. consider copper-based catalysts to be the best catalysts for CO_2_ reduction to hydrocarbon products. They observed using in situ Raman spectroscopy that the peaks at 1071.2 and 1013.8 cm^−1^ are attributed to CO_3_^2−^ and HCO_3_^−^ on Pot-Cu catalysts, while both CO_3_^2−^ and HCO_3_^−^ are present on Pul(3)-Cu catalysts. The Cu−OH signal observed at 529.1 cm^−1^ is more prominent on Pot-Cu than on Pul(3)-Cu, and leads to high peaks at 355.9−366.3, 2030.2−2054.3, and 2091.2−2103.1 cm^−1^, corresponding to Cu−CO stretching, *CO stretching in the low-frequency band (LFB), and *CO stretching in the high-frequency band (HFB), respectively. This indicates that the rough surface and porous structure of Pot-Cu facilitate the enrichment of OH^−^, significantly promoting the dimerization of *CO. It confirms that Cu catalysts with different coordination numbers (CN) exhibit different adsorption behaviors towards *CO, thereby impacting the reaction pathways of CO_2_RR [60].

In the CO_2_RR process, the C≡O stretching mode of adsorbed *CO is one of the most representative characteristics in in situ Raman spectroscopy analysis, whose peak position can reflect changes in adsorption configuration and local reaction environment. Generally, the *CO_atop_ signal is located in the range of approximately 2000–2100 cm^−1^, while the *CO_bridge_ signal is distributed in the range of approximately 1800–1900 cm^−1^. However, the specific peak positions and relative intensities vary with the catalyst surface structure, potential, and reaction conditions. They used TR-SERS to study the potential-dependent spectral characteristics in more detail. The TR-SERS spectra can also be roughly divided into three parts: (1) <700 cm^−1^: vibrations of Cu_2−x_O, Cu-C, and Cu-O(H); (2) 700–1600 cm^−1^: carbonate/bicarbonate electrolyte ions; (3) 2000–2100 cm^−1^: stretching vibrations of adsorbed CO [29,30]. Ruiter et al. used TR-SERS to study the dynamic reconstruction of copper (oxide) electrode surfaces and the adsorption of reaction intermediates during cyclic voltammetry (CV) and pulse electrolysis (PE) processes. When the scan direction is reversed after reaching the maximum anodic bias of +1.0 V vs. RHE, vibrations at ~630, 520, and 400 cm^−1^ are observed, which belong to the Cu_2-x_O surface. These are partially attributed to the Raman-active modes of the Cu_2-x_ lattice (520 cm^−1^, T_2_g) and the chemistry of defect-rich copper oxides (400 and 630 cm^−1^). However, pulsed electrolysis (PE) at different cathodic biases shows that stochastic CO dominates initially, while no CO intermediates are observed after long-term application at low overpotentials. When the cathodic bias is −0.55 V, it leads to the formation of statically adsorbed CO intermediates and a decrease in stochastic CO. This phenomenon reveals the reconstruction of oxide-derived copper electrodes and the formation of CO at low overpotentials [29].

The broad bands appearing in the 2800–3000 cm^−1^ range are attributed to adsorbed alkyl fragments. C−H stretching vibrations of CH_x_ fragments, such as the *CH_2_ symmetric stretching at 2848 cm^−1^, *CH_3_ symmetric stretching at 2874 cm^−1^, *CH_2_ asymmetric stretching at 2904 cm^−1^, and *CH_3_ asymmetric stretching at 2961 cm^−1^ observed on Cu foam electrodes. Given the significant overlap in peak heights within this region and the potential correspondence to multiple structures—such as *CH_3_, *CH_2_, *CH, or oxygen-containing intermediates *OCH_2_CH_3_ C−H modes—this complexity currently precludes definitive determination of whether the reaction is initiated by a single common initial intermediate or involves multiple distinct initial intermediates participating in parallel [61,62]. Li et al. designed Poly(ionic liquid)–metal (PIL-metal) hybrids (Cu@PIL) and introduced metal components Ag or Bi with different CO_2_RR response characteristics on their surface, constructing Cu@PIL@Ag and Cu@PIL@Bi tandem system catalysts. They used in situ Raman spectroscopy to monitor under real electrochemical conditions. At open circuit potential, characteristic peaks related to Cu_2_O and Ag–OH vibration peaks were observed on the surface of Cu@PIL@Ag at 513, 626 cm^−1^ and 290 cm^−1^, and 685 cm^−1^. However, these peaks decay rapidly when the cathodic potential is lower than –0.07 V, indicating that Cu and Ag species are reduced to metallic states and undergo rapid re-oxidation after depolarization. In terms of intermediate identification, *COOH at 406 cm^−1^, *OCO at 554 cm^−1^, and a broadened *CO stretching vibration peak in the range of 2000–2150 cm^−1^ were observed on Cu@PIL@Ag. Their potential-dependent changes reveal that the strong adsorption of *OCO by Ag sites inhibits the formation of HCOOH, while C–C coupling at high overpotentials leads to the disappearance of the *CO signal. In contrast, characteristic peaks of *OCHO at 1283 cm^−1^, 1440 cm^−1^, 1465 cm^−1^ and vibration peaks of *CO_3_^2−^ at 857 cm^−1^, 1394 cm^−1^ were detected on Cu@PIL@Bi, indicating that Bi sites are more inclined to form carbonates and HCOOH. These results show that in situ Raman can effectively reveal the types of intermediates on different metal interfaces, their adsorption strength, and their regulatory effect on product distribution, providing strong support for understanding the structure-performance relationship of Cu-based multimetallic catalysts [63]. Chernyshova et al. used SERS technology to study the key intermediates for ethylene and ethanol formation during CO_2_RR on Cu surfaces, identified the characteristic peaks of asymmetric stretching vibration of CO_2_^−^ at 1540 cm^−1^ and symmetric stretching vibration of Cu–C at 350 cm^−1^ that appear in the range of −0.3 V to −0.9 V and change with potential. The results show that low-coordination Cu defect sites can stabilize CO_2_RR intermediates, thereby maintaining high *CO coverage and promoting C–C coupling. [61]. Lim et al. designed histidine-functionalized Cu for CO_2_RR. Studies via in situ Raman spectroscopy observed Cu_2_O peaks at 519, 629 cm^−1^ and possible carbonate peaks at 1073 cm^−1^, along with weak histidine-related peaks. After applying a cathodic potential, the carbonate peaks disappeared, and histidine-related peaks at 1009, 1259, 1321, and 2079 cm^−1^ were significantly enhanced, with no typical Cu-CO or C≡O vibration peaks detected. This reveals an alternative reaction pathway involving direct interaction between adsorbed histidine and CO_2_ reduction intermediates at higher cathodic potentials [62]. Hong et al. designed a copper-supported iron single-atom catalyst for CO2 electroreduction to generate CH_4_, and combined in situ Raman spectroscopy to reveal the adsorption and conversion process of reaction intermediates. Under CO_2_RR conditions, they observed characteristic peaks at 365 cm^−1^, 1079 cm^−1^, and 535 cm^−1^, belonging to Cu–CO stretching, symmetric stretching of CO_3_^2−^ on Cu surface, and Fe–CO stretching, respectively. They also found that during the potential range from −0.7 V to −1.3 V vs. RHE, the phthalocyanine ring vibration peaks weakened until they disappeared, indicating the breaking of Fe–N bonds, the detachment of phthalocyanine rings, and the formation of Cu–Fe single-atom structures. Further analysis shows that Fe sites have a stronger adsorption capacity for *CO, which can inhibit *CO adsorption on Cu sites, thereby enhancing methane formation activity and selectivity [63].

**Figure 5:**
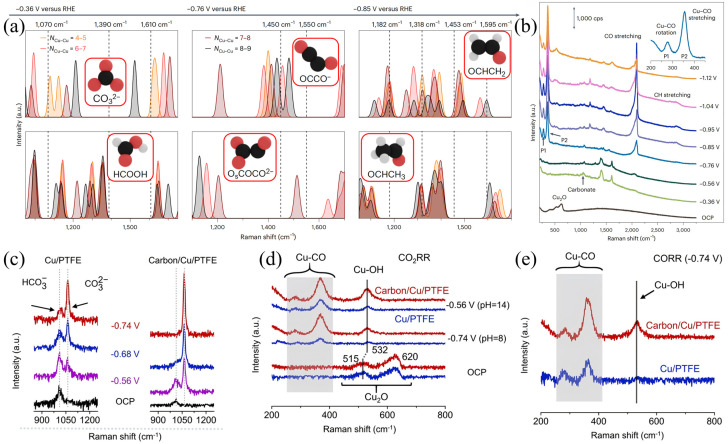
(**a**) DFT−optimized CO_2_RR reaction intermediates and their vibrational fingerprints. (**b**) Raman spectra of an electrochemically treated Cu foil acquired during CO_2_RR for potentials ranging from the open−circuit potential (OCP) to about −1.1 V vs. RHE in a CO_2_−saturated 0.1 M NaClO_4_ electrolyte. cps, counts per second. Reproduced with permission from Ref. [65]. Copyright 2024, Nature Publishing Group. (**c**) In situ Raman spectra obtained from Cu/PTFE (**left**) and carbon/Cu/PTFE (**right**). (**d**) In situ Raman spectra of Cu/PTFE (blue) and carbon/Cu/PTFE (red) during CO_2_RR. The region between 240 and 415 cm^−1^ is shaded. (**e**) In situ Raman spectra collected at different electrodes under CO_2_RR at −0.74 V. Reproduced with permission from Ref. [68]. Copyright 2023, American Chemical Society.

Complex adsorbed intermediates such as C-H, O-CH-O, and C-O can be identified by in situ Raman spectroscopy [64,65]. In Figure 5a, the main reaction intermediates that give rise to the experimentally detected vibrational frequencies are shown. After the reduction of the Cu oxidized phase, *CO_3_^2−^ and *HCOO/*HCOOH species are formed (see the 1070 cm^−1^, 1390 cm^−1^, and 1610 cm^−1^ signals in Figure 5b). These frequencies can be assigned to the *OCCO dimer adsorbed on surface oxygen (Os), which is referred to as the deprotonated glyoxylate OsCOCO_2_^−^. [66,67]. Sakamoto et al. used anisotropic Ag core/SiO_2_ shell nanoparticles as SERS substrates to perform in situ Raman analysis of C–C coupling reactions during CO_2_ reduction. On the CuBr-4PP catalyst, they observed characteristic peaks at 1740 cm^−1^ and 2050 cm^−1^ when the potential was −1.2 V vs. Ag/AgCl, and a peak in the range of 1540–1560 cm^−1^ appeared when the potential was shifted to −2.0 V. However, these peaks did not appear in an Ar atmosphere or on CuBr-12B (Cu_2_(μ-Br)_2_(1,2-bis(diphenylphosphino)benzene)_2_). Combined with CO_2_ labeling experiments, they attributed these signals to C–C coupling intermediates derived from CO_2_, thereby revealing the mechanism of multicarbon intermediate formation between two Cu centers [65]. Rajagopalan et al. attempted to research the scaling relationships between intermediate adsorption energies via in situ Raman spectroscopy and density functional theory, described the unique features found in two-dimensional materials, and indicated that future research focusing on the electrode-electrolyte interface would facilitate the design of high-performance CO_2_RR catalysts [50].

Studies on carbonates in regulating CO_2_RR activity and selectivity have been conducted via in situ Raman spectroscopy [2,69,70]. Jiang et al. systematically compared the interface reaction characteristics of Cu-based catalysts derived from Cu_2_O superparticles and cubes in CO_2_RR. In situ Raman spectroscopy shows that Cu_2_O superparticles exhibit a C–O stretching peak of *CO at 2060–2080 cm^−1^ at −0.65 V vs. RHE, the peak intensity significantly increases at −0.75 V vs. RHE and gradually weakens at −0.95 V vs. RHE, indicating that *CO intermediates have been further converted into multicarbon products such as C_2_H_4_. Meanwhile, it exhibits a significantly stronger adsorbed CO_3_^2−^ symmetric stretching peak at 1070 cm^−1^. Combined with the equilibrium relationship of KHCO_3_ electrolyte, it can be inferred that Cu_2_O superparticles induce a higher local pH during the reaction. This local alkaline environment not only reduces the energy barriers for *CO formation energy barrier and C–C coupling but also inhibits HER, thereby significantly improving the selectivity of C_2+_ products [2]. Wang et al. designed a catalyst-electrolyte interface to improve the electrochemical synthesis of ethylene from CO_2_. Via in situ Raman measurements, as shown in Figure 5c, when the cathodic voltage varies from −0.56 V to −0.74 V, the Raman peak of CO_3_^2−^ becomes more prominent, while the Raman peak of HCO_3_^−^ becomes weaker, indicating that the change in cathodic voltage leads to an increase in pH for C/Cu/PTFE and Cu/PTFE electrodes. Under neutral (pH = 8) or alkaline (pH = 14) conditions during CO_2_RR, the signal intensity of the Cu-CO peaks observed at 293 and 360 cm^−1^ and the Cu–OH peak observed at 532 cm^−1^ on the Cu/PTFE surface is much lower than that on the C/Cu/PTFE surface. As shown in the SERS spectrum in Figure 5d, under cathodic potential, asymmetric catalytic centers (such as Cu_2_O) are not observed on the pure Cu surface. From the in situ SERS results shown in Figure 5e, at −0.74 V, C/Cu/PTFE exhibits higher *CO coverage and stronger Cu−OH signals than Cu/PTFE. The higher surface CO coverage and OH^−^ concentration reduce the C−C coupling energy, thereby enhancing the efficiency of ethylene production [68]. It is thus concluded that the highly alkaline local environment at the interface reduces the energy of the first proton-electron transfer and promotes the formation of ethylene products in CO_2_RR. This further confirms that an appropriate pH facilitates the CO_2_RR reaction towards C_2+_ products [71,72,73].

## 5. Monitoring the Structural and Valence Evolution of Cu-Based Catalysts

In situ Raman spectroscopy has become a mainstream characterization method for studying the CO_2_RR process, which can real-time track the dynamic evolution of catalyst surface structures and the generation of reaction intermediates under real reaction conditions, and especially exhibits unique advantages in analyzing the structure-performance relationship of Cu-based materials [27,28,30,74,75].

**Figure 6:**
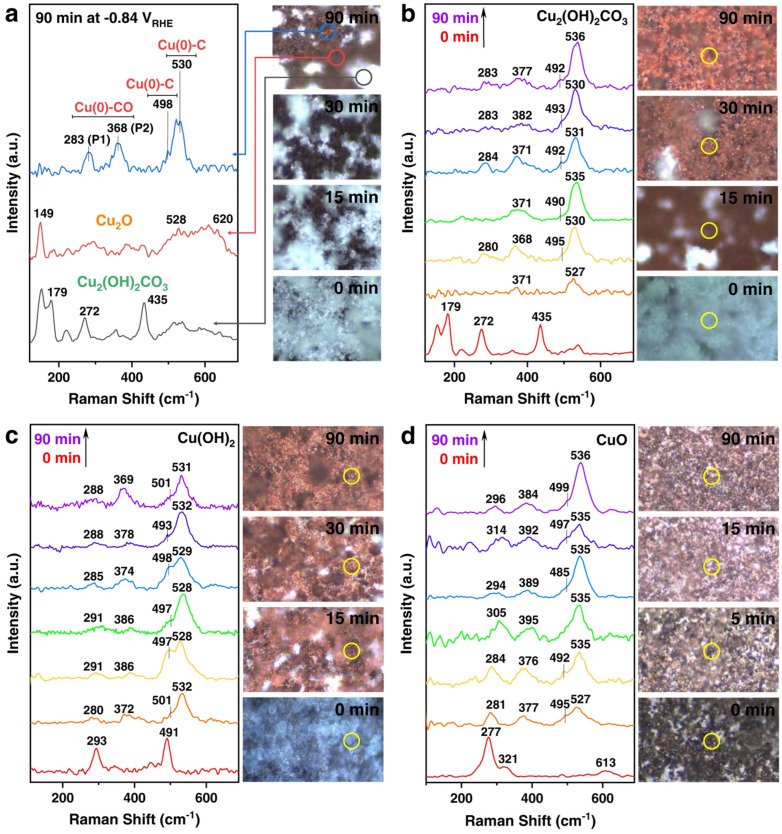
Operando Raman spectroscopy characterizations. (**a**) Time −resolved light microscopy (**right panel**) images of the Cu_2_(OH)_2_CO_3_ electrode surface during CO_2_RR at −0.84 vs. RHE, and the operando Raman spectra (**left panel**) acquired from three different areas (circled in different colors) on the Cu_2_(OH)_2_CO_3_ electrode surface after 90 min of reaction. (**b**) Cu_2_(OH)_2_CO_3_ at −0.84 vs. RHE, (**c**) Cu(OH)_2_ at −1.08 vs. RHE, and (**d**) CuO at −1.16 vs. RHE during CO_2_RR, showing time-resolved operando Raman spectra (**left panel**) and light microscopy images (**right panel**) at their optimum potentials for C_2+_ production. All measurements were conducted in CO_2_−saturated 0.1 M KHCO_3_ electrolyte, and Raman spectra were acquired using a 633 nm laser as the excitation source, with spectra collected every 15 min. Reproduced with permission from Ref. [76]. Copyright 2022, Nature Publishing Group.

**Figure 7:**
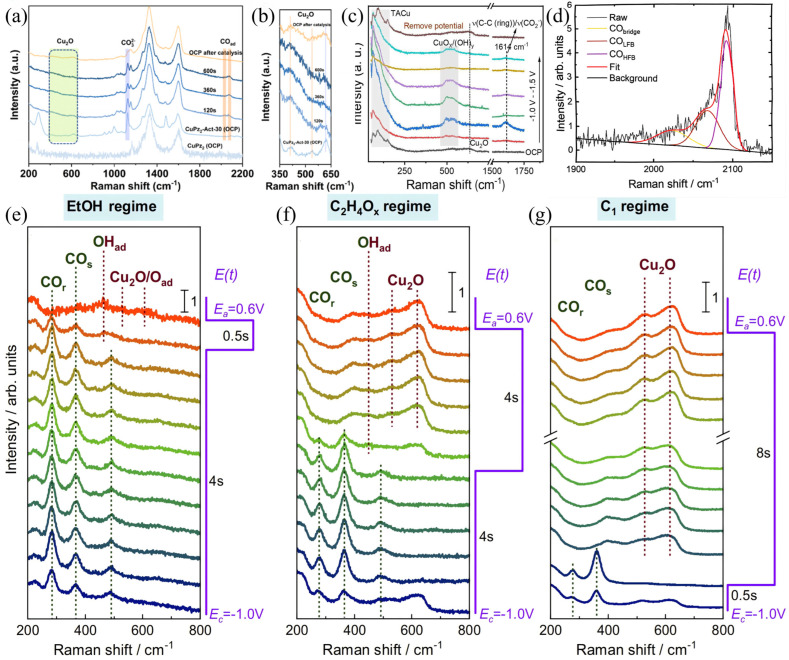
In CO_2_−saturated 0.1 M KHCO_3_ electrolyte, (**a**,**b**) in situ Raman spectra of CuP_z2_, CuP_z2_−Act−30, and CuP_z2_−Act-30 during different reaction durations, as well as the in situ Raman spectrum of CuPz_2_−Act-30 in 0.1 M KCl solution under CO_2_ bubbling conditions. Raman spectra were acquired using a 532 nm laser as the excitation source. Reproduced with permission from Ref. [77]. Copyright 2022, American Chemical Society. (**c**) In situ Raman spectra of TA−Cu during CO2RR in 1.0 M KOH electrolyte. Reproduced with permission from Ref. [75]. Copyright 2023, Wiley−VCH GmbH. (**d**) Exemplary fits of the C−O vibration region of a normalized SERS spectrum. (**e**–**g**) Normalized SERS spectra from bottom to top with highlighted characteristic SERS bands during pulsed CO_2_RR with varying pulse lengths at E_c_ = −1.0 V and E_a_ = +0.6 V, using 785 nm excitation laser. Reproduced with permission from Ref. [78]. Copyright 2024, Nature Publishing Group.

Lei et al. used in situ Raman spectroscopy to evaluate the CO_2_RR performance of three Cu-based catalysts in an H-type cell, and analyzed the gas-liquid products under different applied potentials in CO_2_-saturated 0.1 M KHCO_3_ aqueous solution at room temperature. As shown in Figure 6a, during the CO_2_RR process, Cu_2_(OH)_2_CO_3_, at −0.84 V vs. RHE, in some regions gradually changed from the original turquoise to brown after 90 min (right image). By observing three types of Raman spectra located in different regions on the electrode surface (left image), Cu_2_O corresponds to the red circle area in Figure 6a, with fingerprint Raman bands at 149, 528, and 620 cm^−1^. The blue circle area has three strong bands at 283, 368, and 530 cm^−1^, and a shoulder band at 498 cm^−1^. However, when the negative potential of CO_2_RR is removed, the four bands disappear, and the fingerprint spectrum of Cu_2_O appears, confirming the correlation between the bound intermediates on the Cu surface and them. As shown in Figure 6b, they conducted an analysis in the brown region. At potentials of −1.05 V vs. RHE and −1.2 V vs. RHE, no shiny crystals remained on the electrode surface after 30 min, and the Raman spectrum shows 283, 368, 498 (shoulder), and 530 cm^−1^. As shown in Figure 6c, they conducted a study on Cu(OH)_2_. By comparison, CuO exhibits a faster electroreduction rate. Moreover, the initial black electrode surface completely turned brown within the first 5 min. In Figure 6d, the Raman bands of CuO disappear within 15 min, and the peaks at ~498 cm^−1^ and ~530 cm^−1^ demonstrate that Cu^0^ still dominates the electrode surface. Ultimately, it is shown that when the maximum potential is applied, the three oxidized Cu precursors (bulk and surface) are completely reduced to metallic Cu, and CuO exhibits faster reduction kinetics than the other two [76]. Liu et al. reported a Cu-N coordinated MOF reconstructed to form dispersed Cu/Cu_2_O nanoclusters for the selective reduction of CO_2_ to C_2_H_4_. Figure 7a,b show that they used in situ Raman spectroscopy to detect the Raman absorption peaks of Cu_2_O and the Raman absorption peaks of adsorbed CO for CuPz_2_, CuPz_2_-Act-30, CuPz_2_-Act-30, and post-catalysis CuPz_2_-Act-30 under open circuit potential (OPC). The CuPz_2_ precursor has no Cu_2_O Raman absorption peaks at 412, 528, and 619 cm^−1^, and there is no obvious adsorption for the adsorbed CO peaks. After electrolysis, without exposure to ambient air, the in situ state is obtained after activation of CuPz_2_. Afterwards, due to the influence of Cu_2_O, at open circuit potential, CuPz_2_-Act-30 shows multiple Raman absorption peaks at 412, 528, and 619 cm^−1^. The above data demonstrate that Cu_2_O species are generated after electrochemical activation. When in situ Raman spectroscopy is re-measured at OCP, the Raman absorption peaks of Cu_2_O are enhanced again, and Cu nanoclusters are spontaneously generated along with Cu_2_O during the activation process. It is proven that the key CO intermediates during electrolysis may be adsorbed on Cu_2_O and Cu nanoclusters as active sites, confirming the key role of Cu/Cu_2_O nanoclusters in the formation of C_2_H_4_ [77]. Chen et al. designed a tannic acid (TA) molecule to adaptively regulate the reconstruction of Cu-based materials, enabling them to promote CO2 reduction to C_2_H_4_. In Figure 7c, through in situ Raman analysis, it was found that the TA-Cu spectrum is the same as that of CuTA in the dry state under OCP conditions, with an obvious characteristic peak at 1000 cm^−1^, confirming the stability of CuTA in the electrolyte. When an overpotential is applied, the peaks at 522–670 cm^−1^ shift to 493–531 cm^−1^ attributed to Cu-O, indicating a change in the local Cu-O environment. Moreover, due to the C-H vibration of C_2_H_4_, a new peak appears at 1614 cm^−1^, namely v(C-C(ring))/v(CO_2_^−^). This is beneficial for maintaining Cu in a partially oxidized state, thus providing a strategy for CO_2_ to value-added chemicals [75].

The induced kinetics of surface coverage of hydrogen (H_ad_), hydroxide (OH_ad_), and carbon monoxide (CO_ad_) on Cu-based catalysts are crucial for the fundamental principles of CO_2_RR selectivity changes. However, due to the lack of surface-sensitive operando characterization, determining whether surface Cu oxides exist still poses certain challenges [29,78,79]. Herzog et al. used TR-SERS to track the dynamic structural evolution of prereduced Cu_2_O nanocubes and changes in adsorbed species during the CO_2_RR process. Through spectral analysis under ethanol regime (EtOH mode, Figure 7e), ethylene/acetaldehyde regime (C_2_H_4_O_x_ mode, Figure 7f), and C_1_ regime (C_1_ mode, Figure 7g), they revealed the spatiotemporal distribution characteristics of Cu_2_O phases, CO adsorption configurations, and surface hydroxyl (OH_ad_) species in different product pathways. The results show that anodic pulses promote the growth of Cu_2_O layers, the rate of which is significantly slower than the reduction/desorption process under cathodic pulses, and exhibits the most significant continuous growth trend under conditions for C_1_ products. As shown in Figure 7d, the CO Raman region at 1900–2150 cm^−1^ shows that the *CO_atop_ (low frequency of approximately 2065 cm^−1^ and a high frequency of approximately 2095 cm^−1^) dominate under conditions for C_2+_ products, whereas the *CO_bridge_ (at approximately 2030 cm^−1^) is significantly enhanced under conditions for C_1_ products, which reveals the correlation between different adsorption configurations and C–C coupling activity. OH_ad_ related bands (370–380, 520–540 cm^−1^) are significantly enhanced at the initial stage of anodic pulses, accompanied by peak position shifts caused by the Stark effect due to electric field changes generated by potential switching, indicating that they may act as intermediate species for Cu_2_O formation. These data confirm that nanocrystalline Cu_2_O domains can be generated at the end of anodic pulses, accompanied by a small amount of highly disordered Cu(II) species. This work systematically reveals the temporal evolution of surface redox cycles and intermediate configurations of cuprous oxide nanocubes under CO_2_RR conditions, providing direct spectral evidence for understanding product selectivity regulation [78].

**Figure 8:**
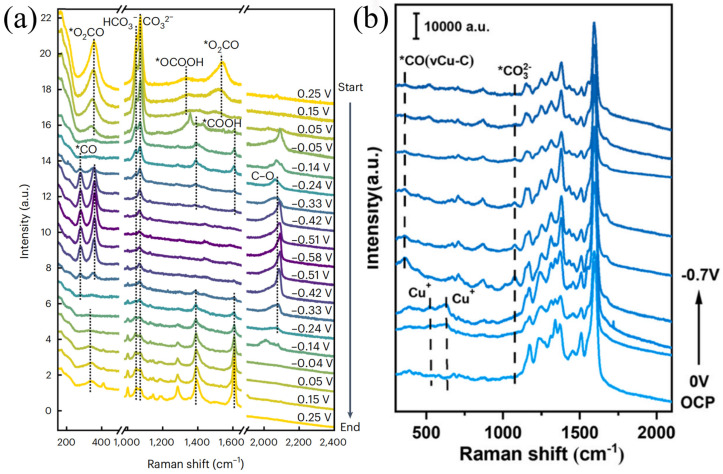
(**a**) Potential-dependent molecular-scale structure of Cu(100) in CO_2_−saturated 0.1 M KHCO_3_. In situ SERS spectra of Cu(100) were recorded during the stepwise decrease of the potential from 0.25 V to −0.58 V and the subsequent increase back to 0.25 V (* denotes molecules adsorbed on the Cu surface). Raman spectra were acquired using a 633 nm He−Ne laser (17 mW, Renishaw RL633) as the excitation source. Reproduced with permission from Ref. [80]. Copyright 2023, Nature Publishing Group. (**b**) In situ Raman spectra of TpBpy−Cu−10 during CO_2_RR at potentials ranging from the open circuit potential (OCP) to −0.7 V vs. RHE in 0.1 M KHCO_3_. Raman spectra were acquired using a Renishaw in Via Reflex spectrometer with a 532 nm laser as the excitation source. Reproduced with permission from Ref. [81]. Copyright 2025, Elsevier.

**Figure 9:**
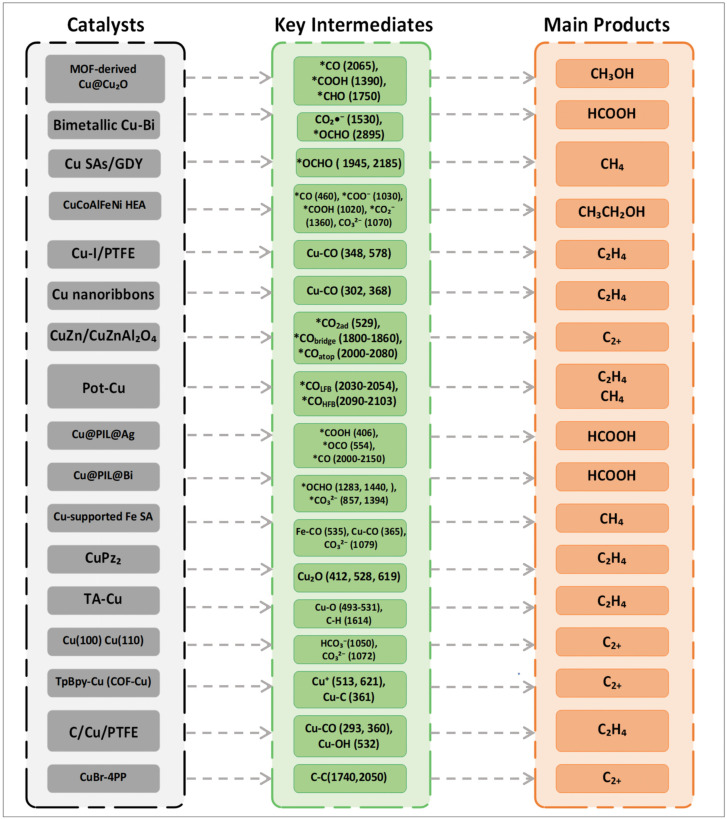
Relationship among Cu–based catalyst structure, reaction intermediates, and product selectivity in CO_2_ reduction.

The potential kinetics study on inducing structural changes of Cu catalysts in CO_2_RR is of great significance for guiding catalysts to selectively generate multi-carbon products. Surface reconstruction plays an important role in the activity and selectivity of CO_2_RR [80,81]. Amirbeigiarab et al. used a SERS system to study the CO_2_RR process of Cu(100) electrodes in a CO_2_-saturated 0.1 M KHCO_3_ electrolyte. Prior to the experiment, pretreatment was performed on the copper electrode via repeated redox to ensure the complete reduction of the Cu surface, and Raman spectra were collected during the gradual application of cathodic potential. As shown in Figure 8a, there are strong Raman peaks at 360 and 1540 cm^−1^, corresponding to bidentate carbonate (*O_2_CO) adsorbed on or near the Cu(100) surface. The peaks at 1050 and 1072 cm^−1^ correspond to HCO_3_^−^ and CO_3_^2−^, respectively. The weak peak at ~1000 cm^−1^ may originate from dissolved bicarbonate or adsorbates related to formic acid/formate. They also pointed out that the weakening of the peaks at 360 and 1540 cm^−1^ may also be related to the reduction process of oxides such as CuCO_3_. In the potential scan, as the potential decreases from 0.25 V to −0.05 V, the original carbonate peaks at 360 and 1540 cm^−1^ gradually weaken, and new peaks at 1390 and 1610 cm^−1^ appear at −0.05 V, which correspond to the symmetric and asymmetric vibrations of adsorbed carboxylate ions or carboxyl groups, respectively. These species are considered precursor intermediates of *CO in the CO_2_RR process. When the potential drops below −0.33 V, these carboxylate-related peaks disappear, indicating that their formation has a certain potential dependence. Meanwhile, both high wavenumber (CO atop, ~2090–2100 cm^−1^) and low wavenumber (CO bridge, ~2030 cm^−1^) regions appear, showing a reversible conversion trend: under low overpotential, bridge-bonded CO with low coverage dominates, while under high overpotential, it gradually converts to atop CO structures with high coverage and higher activity. In addition, below −0.04 V, a broad peak appears at 2900 cm^−1^ in the Raman spectrum, attributed to the C–H stretching vibration mode, which is related to the formation process of carboxylates; this peak remains in the positive potential scan, indicating that some intermediates have irreversible adsorption on the surface. It reveals the key adsorbed species and their conversion pathways on the Cu(100) surface during CO_2_RR, indicating that the configuration transition of *CO is closely related to C–C coupling activity, and some carboxylate intermediates have strong surface stability in the reaction [80]. Yu et al. utilized the nitrogen-containing functional groups of COF-TpBpy to design a Cu-COF catalyst (Tp represents 2,4,6-triformylphloroglucinol; Bpy represents [2,2′-bipyridine]-5,5′-diamine) coordinated with copper acetate, and conducted in situ Raman analysis, obtaining the evolution of TpBpy-Cu catalyst and the progress of CO_2_RR (Figure 8b). The overpotential of Cu+ at 513 and 621 cm^−1^ is relatively low; Cu^2+^ is reduced to Cu^+^, and as the potential shifts to more negative values, the corresponding peaks of Cu^+^ disappear, with Cu^+^ further reduced to Cu^0^. Meanwhile, a Cu-C stretching vibration appears for Cu-adsorbed CO at 361 cm^−1^, confirming that the active sites are in situ generated Cu nanoparticles. It reveals the structural transformation from TpBpy-Cu to Cu nanoparticles during the CO_2_RR process [81]. As shown in Figure 9, Cu-based catalysts with different structures (single-atom, bimetallic, MOF-derived, etc.) can regulate the formation of key intermediates such as *CO, *COOH, Cu-CO, and *OCHO. The difference in intermediates directly determines product selectivity: *CO/*COOH-type intermediates tend to generate CH_3_OH. *OCHO is conducive to HCOOH. Cu-CO-type intermediates often promote C_2_H_4_ or C_2+_ products, and CO in different adsorbed states can lead to CH_4_. This demonstrates the correlation between catalyst structure, intermediates, and product selectivity, and plays a key role in achieving efficient resource-oriented conversion of CO_2_, as well as in carbon emission reduction and green chemical synthesis.

## 6. Conclusions and Outlook

This paper systematically reviews the research progress of in situ Raman spectroscopy in CO_2_RR over Cu-based catalysts, focusing on discussing the unique advantages of this technique in revealing catalyst structure evolution, active site transformation, and identification of key reaction intermediates. As shown in Table 1, in situ Raman spectroscopy not only provides molecular-level information in analyzing the CO_2_RR mechanism of Cu catalysts but also lays a solid experimental foundation for the rational design of high-efficiency catalytic systems for C_2+_ products. A large number of studies have shown that Cu derivatives are rapidly reduced and undergo surface reconstruction under CO_2_RR conditions, generating metallic Cu or Cu^+^–Cu^0^ composite structures with specific crystal facets and nanoscale morphologies; these structures can effectively promote C–C coupling and improve the selectivity of C_2+_ products. With the help of signal enhancement strategies such as time-resolved SERS, researchers can track the adsorption and transformation behavior of transient intermediates such as *CO, *OCCO, *COOH, *CH_2_CHO and *CHO on a sub-second scale, and analyze the influence of local pH, interface electrolyte composition, etc., on reaction pathways.

However, technical challenges such as weak signal intensity, laser-induced sample degradation, and background fluorescence interference still exist, limiting the widespread application of in situ Raman under higher current densities and industry-relevant conditions. Although SERS has significantly enhanced the signal collection capability on Cu-based catalysts, there are still blind spots in monitoring some intermediates with low coverage or extremely fast conversion. In recent years, although in situ Raman spectroscopy was initially mainly applied to H-type electrolytic cells with mA-level current density, an increasing number of studies have expanded its application to gas diffusion electrode (GDE) and membrane electrode assembly (MEA) systems, investigating catalyst interface states and intermediate evolution at current densities ranging from hundreds of mA to A levels, thus being closer to industry-relevant conditions [85,86]. Wang et al. designed a membrane-based GDE/PE in situ Raman cell to investigate Pt/Nafion interfaces. The study identified weakened interfacial hydrogen bonding, restricted fluorocarbon chain mobility, and a looser electric double layer, as reflected by characteristic ν(Pt–O) and ν(CF_2_) vibrations. These findings highlight the critical role of Nafion dynamics in facilitating gas transfer while minimizing ionomer degradation, thereby extending the applicability of in situ SERS to membrane-based electrochemical environments [85]. Therefore, it is necessary to improve the detection sensitivity and frequency coverage range of in situ Raman in the future. This can be achieved by developing multi-wavelength lasers and broadband Raman collection technologies to realize continuous detection from low-wavenumber lattice vibrations to high-wavenumber C–H and C–O modes, thereby capturing more information related to C_2+_ formation.

Moreover, a single spectroscopic method is difficult to fully describe the structure-chemical state evolution of Cu catalysts during the CO_2_RR process. Coupling in situ Raman with multiple techniques such as XAS, synchrotron radiation Fourier transform infrared (SR-FTIR), and electrochemical mass spectrometry (EC-MS) to reveal the multi-scale structure-performance relationships of Cu-based catalysts enables synergistic analysis at atomic, molecular, and mesoscale levels, and reveals the intrinsic correlations between changes in Cu^0^/Cu^+^ ratio and intermediate stability.

By interface regulation and local reaction environment design and constructing hydrophobic surfaces, local alkaline microenvironments, or specific coordination chemistry, Cu^+^ species can be stabilized and *C–C coupling efficiency can be improved. In the future, in situ Raman can be used to real-time evaluate the impact of different interface regulation strategies on intermediate coverage and product distribution, reversely guiding the molecular-level design of high-efficiency Cu-based catalysts. Moreover, with the continuous development of in situ Raman devices suitable for high-flow CO_2_ supply and strongly alkaline electrolyte environments, it is expected to reveal the reaction kinetics and deactivation mechanism of Cu-based catalysts under real operating conditions. With the continuous improvement of detection sensitivity, the in-depth integration of multi-technical characterization, and the breakthrough of operando methods, it is expected to achieve a comprehensive and accurate description of the structure-activity relationship of Cu-based catalysts in the future, providing solid scientific support for CO_2_ resource utilization under the goal of carbon neutrality.

## Figures and Tables

**Table 1:** Assignments of Raman peaks over Cu catalysts for CO2RR.

Raman Shift (cm^−1^)	Assignments	Reference
Cu_2_O	~149, 218–224, 406–427, 510–528, 610–650	[27,53,72,76,77,79,82,83]
CuO	285–290, 590–601, 621–628	[58,59,82,83]
Cu(OH)_2_	280–292, 475–483, ~550	[36,37]
Cu-CO frustrated rotation	280–284, ~291	[57,59,76,79,80]
Cu-CO stretching	~348, 350–375, ~380	[2,22,31,40,46,57,60,64,65,79,80,82,84]
Cu-OH adsorption vibration	~424, 520–530, 700–706	[2,22,37,60,72,84]
*CO_3_^2−^	1050–1056, 1060–1068, 1070–1079, ~1392	[2,27,37,47,60,61,64,65,78,80,81,84]
*HCO_3_^−^	1000–2000, 1335–1366	[2,26,27,37,42,60,65,72,80]
V_s_CO_2_^−^ V_as_CO_2_^−^	1324–1360, 1524–1554	[27,44,47,84]
*H_2_O	1635–1647	[26,81]
*CO_atop_	2000–2100	[26,34,39,42,46,56,58,59]
*CO_bridge_	1800–1900	[2,34,40,42,46,58,59,78]
C-H stretching	2700–3100	[26,42,65,80,82,84]
V Cu-C[Cu-C] stretching vibration	342–361	[27,61,81,84]
*OCCO	~1540, 2088–2094	[27,58]
*COOH	1370–1383, 1390–1443	[26,38,40,43,59,77,81]
*C-O stretching	1000–1070	[37,38]
*OCCOH	1205–1260, 1520–1645	[26,38,58,59]
C-OH stretching	1010–1032, ~1262	[82,84]
hindered rotation of adsorbed CO	275–290	[22,65,76,82]
C-C stretching	~934, ~1453	[65,84]
C≡O stretching	1800–2100, 1900–2200, ~1700, ~2400	[22,40,56,57,61]
CuO_x_	~290, 345–400, 450–700	[22,65]
OH_ad_	370–380, 450–490, 520–540	[78,79]
CO_LFB_	2030.2–2054.3, 2060–2065	[60,78,79]
CO_HFB_	2090–2103.1	[60,78,79]

## Data Availability

No new data were created or analyzed in this study.
